# Large-scale coupling of prefrontal activity patterns as a mechanism for cognitive control in health and disease: evidence from rodent models

**DOI:** 10.3389/fncir.2024.1286111

**Published:** 2024-04-04

**Authors:** Ignacio Negrón-Oyarzo, Tatiana Dib, Lorena Chacana-Véliz, Nélida López-Quilodrán, Jocelyn Urrutia-Piñones

**Affiliations:** ^1^Instituto de Fisiología, Facultad de Ciencias, Universidad de Valparaíso, Valparaíso, Chile; ^2^Programa de Doctorado en Ciencias Mención en Neurociencia, Facultad de Ciencias, Universidad de Valparaíso, Valparaíso, Chile

**Keywords:** cognitive control, prefrontal cortex, brain oscillations, functional connectivity, rodent models

## Abstract

Cognitive control of behavior is crucial for well-being, as allows subject to adapt to changing environments in a goal-directed way. Changes in cognitive control of behavior is observed during cognitive decline in elderly and in pathological mental conditions. Therefore, the recovery of cognitive control may provide a reliable preventive and therapeutic strategy. However, its neural basis is not completely understood. Cognitive control is supported by the prefrontal cortex, structure that integrates relevant information for the appropriate organization of behavior. At neurophysiological level, it is suggested that cognitive control is supported by local and large-scale synchronization of oscillatory activity patterns and neural spiking activity between the prefrontal cortex and distributed neural networks. In this review, we focus mainly on rodent models approaching the neuronal origin of these prefrontal patterns, and the cognitive and behavioral relevance of its coordination with distributed brain systems. We also examine the relationship between cognitive control and neural activity patterns in the prefrontal cortex, and its role in normal cognitive decline and pathological mental conditions. Finally, based on these body of evidence, we propose a common mechanism that may underlie the impaired cognitive control of behavior.

## 1 The prefrontal cortex and cognitive control of behavior

### 1.1 Cognitive control of behavior

Animals are immersed in complex and challenging environments. To ensure its survivance and wellbeing, they are able to implement a wide repertoire of adaptive behavioral responses. Some challenges can be solved by the implementation of rapid and simple stimulus-response behaviors, as for example, escape from a predator, or finding a shelter during a sudden natural disaster. Since these behavioral responses are automatically implemented once the stimulus is detected, they allow immediate and fast adaptation (Graybiel, 2008; Buzsáki et al., 2014). However, given that are triggered by particular stimulus, these responses are rigid, stereotyped and lack of voluntarily control, which makes these cognitive operations restricted to be extrapolated to other surrounding events (Buzsáki et al., 2014).

On the other hand, some challenges are directed to the obtention of non-immediate goals, which cannot be successfully solved by stimulus-response behavior (Engel et al., 2001). Of special relevance are those in which current circumstances are new, unknown, or under constant change, and therefore, there is a significant possibility that expectations cannot be accomplished. Under these circumstances, animals require to implement more sophisticated cognitive processes in which acquired information and behavioral responses are constantly updated and accommodated according to current internal (expectations) and external (environmental) conditions. This adaptive guidance and organization of behavioral responses according to current and prospective circumstances in a goal-directed manner is known as cognitive control of behavior (Miller, 2000). It has been postulated that impairment of cognitive control is the core of several normal and pathological mental declines, which is manifested as the reduced ability to implement goal-directed adaptive behavioral responses (Diamond, 2013). Therefore, the understanding of the neurophysiological bases of cognitive control is tremendously relevant not only for the treatment of recovery of mental health, but also as a preventive strategy to ensure and promote well-being. In this article, we review the neurophysiological mechanism that allows the implementation of cognitive control in rodent models to integrate this evidence with findings in normal and pathological conditions observed in humans.

Cognitive control is directed toward the achievement of goals. Therefore, it is voluntarily implemented and self-generated, as it can be implemented without relying on external cues. These goals are commonly based on expectations derived from previous experiences (Miller, 2000; Engel et al., 2001; Diamond, 2013; Buzsáki et al., 2014). Hence, it requires the generation of internal models of environmental conditions extracted from commonalities found across previous experiences (Schlichting and Preston, 2015). Optimal behavioral outcomes are predicted by contrasting and updating the stored internal model (i.e., memory) with the current circumstances, by which plans and strategies emerges from covert internal computations and heuristic processes oriented to goal achievement (Redish, 2016). Thus, even when subjects face novel and ambiguous conditions, successful behavioral responses can be implemented through the interpolation or extrapolation of past and present environmental patterns. As a result, cognitive control is prospective, probabilistic, and generalized (Buzsáki et al., 2014). Given that environmental conditions are far from being stable over multiple temporal scales, cognitive control requires the transient integration of different levels of information. This allows continuous updating of covert computations and behavioral responses (Helfrich and Knight, 2016). This feature allows animals to respond in robust and flexible ways to the continuously fluctuating environment in a goal-directed manner (Buzsáki et al., 2014). This ability, known as behavioral flexibility (Diamond, 2013) is considered one of the hallmarks of cognitive control (Mikhalevich et al., 2017). Loss of behavioral flexibility may lead to profound consequences, manifested as the execution of perseverative and maladaptive behavioral responses, a feature observed in several situations that compromise well-being and mental health, including cognitive decline during aging (Richard’s et al., 2021), mood disorders (Kashdan and Rottenberg, 2010) and schizophrenia (Waltz, 2017), among others (Diamond, 2013; Uddin, 2021).

Cognitive control requires the coordination of several simultaneous neural processes. For example, it involves the integration (binding), interaction and “on-line” temporal maintenance of multiple levels and modalities of information. Computational processes as the evaluation and weighing the relevance of these modalities of information, its comparison with similar occurrences in the past, and the generation of heuristics and prospective responses are simultaneously implemented (Buzsáki et al., 2014). These processes are supported by the synchronization and coordination of several discrete cognitive sub-processes known as “executive functions,” such as focused attention (selecting relevant information), working memory (temporal maintenance of relevant information), recall of experiences (memory recall), inhibitory control, valence interpretation, establishment of stimulus-response associations, goal-setting, strategy implementation, error monitoring, and decision-making (Diamond, 2013). Thus, cognitive control depends on the simultaneous and dynamical cooperation among multiple parallel computations and sources of information.

Several behavioral paradigms in rodent models have been designed to evaluate neural operations related to cognitive control. For example, focused attention and impulse control is often evaluated by 5-choice serial reaction time test (Asinof and Paine, 2014), working memory and decision making are evaluated by delay-match to sample test (Dudchenko, 2004), and inhibitory control can be evaluated by extinction of conditioned-fear (Chang et al., 2009). However, these tests allow the evaluation of a single and discrete cognitive function and are normally based on a particular sensory modality, restricting the evaluation of multiple parallel processes, as occurs during cognitive control. Also, several of these tests are considered of low ethological validity, as they are far from real life circumstances (Shemesh and Chen, 2023). Thus, despite their undeniable value, these tests have remarkable limitations to study neural operations associated to cognitive control.

On the other hand, behavioral paradigm involving goal-directed spatial memory, as the Morris-water maze test, the Barnes maze test, cheese-board test, and radial-arm maze test (Redish, 2016) offers several advantages to study neural processes supporting cognitive control. These tasks are based on learning and memorizing the spatial locations of relevant places in the environment (Ito, 2018). Spatial memory tests are ethologically valid, as it reproduces challenges commonly faced in the natural environments of rodent and primates, including humans (d’Isa and Gerlai, 2022). Given that spatial memory is not acquired in a single trial but it requires the repeated exploration of relatively fixed environment, they allow to evaluate memory generalization and strategy progression along the learning process (Ruediger et al., 2012; Richards et al., 2014). To accomplish the task, animals require to integrate several sets of information as multisensory (visual, olfactory, vestibular), spatial-temporal, emotional valence (aversive and appetitive), and self-motion. Additionally, spatial learning process requires the implementation of discrete executive functions, as extraction of rules and contingences, online maintenance of relevant information (working memory), and decision-making, which can be easily evaluated on spatial memory tasks. Thus, several parallel perceptual and cognitive processes can be evaluated in goal-directed spatial memory tests. Moreover, through a detailed behavioral analysis, they also allow the evaluation of more sophisticated cognitive operations, such as strategy switching (Rich and Shapiro, 2009), vicarious behavior (Redish, 2016), planning (Dragoi and Tonegawa, 2011), path integration (Collett and Graham, 2004), or even imagination (Lai et al., 2023) and deliberation (Blumenthal et al., 2011). Importantly, with slight modifications, it is possible to evaluate behavioral flexibility (Hamilton and Brigman, 2015). For example, in spatial set-shifting tasks, the rule is changed (i.e., navigate using egocentric cues instead of allocentric cues). Similarly, in spatial reversal-learning tasks, the spatial position of the goal is changed after several training sessions, allowing the evaluation of goal-directed adjustment of behavioral. And finally, the neural systems involved in goal-directed spatial memory are relatively well known (Buzsáki and Moser, 2013). Therefore, spatial memory testing in rodent models, though well-designed paradigms, together with a detailed behavioral analysis, is an appropriate behavioral tool for the study of neural mechanisms involved in cognitive control (Pezzulo et al., 2014).

### 1.2 The role of the prefrontal cortex in cognitive control of behavior

The prefrontal cortex (PFC) is the association cortex localized in the frontal lobe of mammals (Carlén, 2017). Lesion and functional studies demonstrate the relevance of the mPFC in several cognitive operations required for goal-directed adaptation, which has led to the proposal that the main and single prefrontal function is “to structure the present to serve the future” (Fuster, 2001, 2008). Therefore, the PFC is considered the main structure supporting cognitive control of behavior (Miller, 2000).

Early studies defined the PFC as the area of the frontal pole that do not evokes motor responses to electrical stimulation (Uylings et al., 2003). In humans, the PFC constitutes a 30% of the entire cortical area. It can be subdivided following cytoarchitectonic criteria into the dorsolateral PFC, ventrolateral PFC, rostral parts of the orbitofrontal PFC and frontal pole. Other regions, such as the caudal orbitofrontal PFC, the anterior cingulate cortex and the ventromedial PFC are also included (Haber et al., 2022). Although rodents do not possess anatomical features of the primate PFC (Carlén, 2017), the rodent medial-PFC (mPFC) subserves a range of cognitive and behavioral processes homologous to those mediated by the primate PFC (Uylings et al., 2003; Carlén, 2017). However, the rodent mPFC has been controversial to define (Carlén, 2017; Laubach et al., 2018). Initially, the rodent mPFC was described as prefrontal areas connected with the mediodorsal nucleus (MD) of the thalamus (Rose and Woolsey, 1948). Later, the homology between the PFC from primates and rodents was established using several criteria, as the cytoarchitecture (presence of granular cortex in the frontal pole), the pattern of specific connections (reciprocal connection with MD), the functional behavioral and electrophysiological properties (similar impairments to prefrontal lesions and comparable activity patterns, as the presence of “delay cells”) and the embryological development (late ontogenetic development) (Uylings et al., 2003). Most of these features are shared between the primate PFC and rodent mPFC. However, taking into account that rodent mPFC is agranular (layer IV is not present), the MD also projects to areas non-related to rodent mPFC (Donoghue and Wise, 1982) and the differences of spatial arrangement between rodent and primate PFC (the rodent mPFC lies next to the allocortex) has led to the view that the primate and rodent PFC are not homologues, but these areas emerged differentially during evolution to accomplish class-common behavioral need, (i.e., the cognitive control of behavior) (Carlén, 2017; Hanganu-Opatz et al., 2023). Also, through comparative studies of gene expression patterns, it has evidenced differences between homologous cortical areas in humans and rodents (Bernard et al., 2012; Zeng et al., 2012). However, recent data based on clustering of internal microcircuitry (Harris et al., 2019) and large-scale hierarchical gradients (Fulcher et al., 2019) show that the rodent and primate PFC have similar laminar-gene expression, cell density and local and large-scale connectivity. Indeed, it was found a high conserved expression pattern of orthologous genes between the human and mouse PFC (Chen et al., 2016). Thus, despite this issue is a current matter of discussion, these data support the idea of “functional homologues” between rodent and primate PFC.

From the behavioral perspective, early studies showed that prefrontal lesions in humans led to the inability to override prepotent responses, which manifested as an impairment in the organization of behavior (Bechara et al., 1994; Damasio, 2006). Detailed behavioral analysis have revealed that prefrontal lesions in humans led to impairments in several executive functions that support cognitive control, as working memory, setting, sustained attention, inference control, decision making, inhibitory control, planning, and strategy implementation (Milner, 1963; Eslinger and Damasio, 1985; Chao and Knight, 1995; Bechara et al., 1998; Burgess, 2000). Importantly, studies developed by Milner (1965) showed that prefrontal lesioned patients showed deficits in spatial mazes, which appeared to be non-spatial in nature but had deficits in the correct strategy to solve the mazes, showing perseverative and impulsive behaviors. Behavioral flexibility is one of the strongest cognitive functions impaired by prefrontal lesions (Wegener and Stamm, 1966). This idea is supported by functional neuroimaging studies in humans showing that behavioral flexibility is associated with the activation of the PFC (Remijnse et al., 2005; Boehme et al., 2017; Uddin, 2021). An extensive body of evidence shows that lesions in the rodent mPFC impairs several executive functions similarly as lesions of human PFC. For example, lesions on the rodent mPFC produced deficiencies in working memory (Ragozzino and Kesner, 2001), focused attention (Kahn et al., 2012), decision-making (Croxson et al., 2014), strategy switching (de Bruin et al., 2001; Floresco et al., 2009) and inhibitory control (Brockett et al., 2022). Evidence from spatial memory tasks has shown that the mPFC supports strategy progression during spatial learning (de Bruin et al., 1997). Notably, the mPFC seems to be required for memory generalization in spatial memory tasks (Richards et al., 2014). Similarly as humans, lesions of the rodent mPFC strongly impair behavioral flexibility [extensive review in Hamilton and Brigman (2015)]. This body of evidence supports the idea of the rodent mPFC as a “functional homologue” to the human PFC.

The rodent mPFC has been subdivided into several areas following cytoarchitectonic criteria. However, different delineations and nomenclatures have been established over the years (Le Merre et al., 2021). The most accepted delineation subdivides the mPFC into three main sections: the infralimbic mPFC (IL), the prelimbic mPFC (PL), and the anterior cingulate cortex (ACC) (Laubach et al., 2018). Some authors include the orbitofrontal cortex (OFC) as part of the rodent mPFC, whereas the ACC is sometimes excluded from the prefrontal criteria (Carlén, 2017; Le Merre et al., 2021). Furthermore, some authors include areas of the dorsal portion of the frontal poles, as the secondary motor cortex (M2), also known as the frontal orienting field (FOF), second frontal area (Fr2), or medial agranular cortex (AGm) as part of the mPFC (Barthas and Kwan, 2017). This region receives afferents from the MD; however, electrical stimulation evokes motor response (Donoghue and Wise, 1982). These discrepancies show the difficulty of demarcate the mPFC in rodents (Carlén, 2017). Thus, the PL and IL are considered the “core” of the rodent mPFC.

Functional differences have been found between these prefrontal subdivisions: for example, during fear conditioning, the PL is involved in the expression of conditioned fear, whereas the IL is required for its extinction (Sierra-Mercado et al., 2011). This has led to a dorsal-to-ventral parcellation of the mPFC, in which the dorsal portion (ACC, PL) is associated with limbic and cognitive operations, whereas the ventral portion (the IL) is associated with visceral and autonomic functions (Vertes, 2004; Peters et al., 2009). However, neuronal firing in the PL and IL seems to represent similar behavioral elements in spatial tasks (Baeg et al., 2003; Hok et al., 2005; Rich and Shapiro, 2009), suggesting similar computing properties between these areas. Indeed, current evidence has challenged this parcellation of the mPFC. For example, diverse prefrontal neuronal populations with differential and opposed representational features coexist in the same prefrontal region (Ye et al., 2016). Analysis of wiring and molecular properties did not found differences between the prefrontal subdivisions (Ye et al., 2016; Ortiz et al., 2020), and behavioral evidence non-related to fear conditioning and extinction suggests functional similarities between the PL and IL (Riaz et al., 2019). Further, using optogenetic tools, it has been shown that activation of PL enhanced fear extinction, whereas inactivation of IL has no effect on extinction, challenging the classical roles of PL and IL (Do-Monte et al., 2015; Marek et al., 2018). Thus, considering the dense reciprocal connectivity between the PL and IL (Hoover and Vertes, 2007; van Aerde et al., 2008), it is possible that these structures work together as a single and unified processing system (Le Merre et al., 2021).

The rodent mPFC is composed by excitatory pyramidal neurons (PN; 80–90% of the total population) positioned in cortical layers II/III and V/VI (Riga et al., 2014) and GABAergic inhibitory neurons (IN; 10–20% of the total population) subdivided into different neuronal sub-types distributed across all cortical layers (Kawaguchi and Kubota, 1997). Importantly, the rodent mPFC is agranular, lacking the layer IV (Uylings et al., 2003). While PN are the main target of afferents from distributed neural systems and constitutes the output from the mPFC to other cortical and subcortical structures (Elston, 2003), IN synapse predominantly locally with PN [but not exclusively, see (Cho et al., 2023)], thus controlling and synchronizing the input and outputs of the prefrontal network (Riga et al., 2014). Among PN, intratelencephalic neurons (IT, neurons projecting to other cortical areas) are distributed between layer II to VI, pyramidal tract PN (PT, neurons projecting to subcortical nucleus) are located in layer V, and corticothalamic PN (CT, which project to thalamus) are located in layer V and IV (Anastasiades and Carter, 2021). Despite the local circuitry of the mPFC has not been studied in detail, it has been suggested that it shares an organization similar to other frontal cortices (Anastasiades and Carter, 2021). In this local connectivity, PN of layer II/III send descending projections to PN in layer V, which also send ascendent projections to layer II/III. Lateral projections are particularly strong in the mPFC, as robust connection exist between PN of layer II/III and between PN of layer V (Anastasiades and Carter, 2021). Indeed, the mPFC is the cortical region of the highest proportion of feedback projections (Le Merre et al., 2021). This high internal excitatory connectivity may be relevant for local neural operations performed in the mPFC. On the other hand, GABAergic IN are mainly subdivided in parvalbumin (PV) and somatostatin (SOM) expressing neurons (Kawaguchi and Kubota, 1997). PV cells synapse preferentially at the soma and axons of PN, contributing to feedforward inhibition that controls signal transmission, whereas SOM cells inhibits the dendrites of PN, providing feedback inhibition (Anastasiades and Carter, 2021). Importantly, cortical and subcortical areas projecting to the mPFC also synapse local IN, which are activated before projecting neurons (Anastasiades et al., 2018). Thus, considering that IN are relevant for the synchronization of neural populations and the emergence of neural activity patterns (see below), the feedback and feedforward inhibition triggered by local and long-range activity may support complex network dynamics in the mPFC.

The mPFC is structurally positioned between the perception and execution of actions, allowing the integration of perceptual information about the current context required for the execution of appropriate behavioral responses (Fuster, 2001). The mPFC receives projections from structures processing sensory, motivational, contextual, spatial, temporal, and internal information necessary to update internal representations (Fuster, 2001; Euston et al., 2012). Indeed, among cortical regions, the mPFC receive projections from the largest number of brain areas (Le Merre et al., 2021). Simultaneously, the mPFC projects to distributed associative, sensory, motor, neuromodulatory, and visceral brain systems to generate and modulate behavioral responses (Vertes, 2004; Gabbott et al., 2005; Euston et al., 2012). One of the most relevant structures innervating the mPFC is MD of the thalamus, which has been considered as a definitory feature of the mPFC (Vertes et al., 2007; Delevich et al., 2015; Ketz et al., 2015; Bolkan et al., 2017; Shepherd and Yamawaki, 2021). Afferences from the MD project to superficial layers of the mPFC (Anastasiades and Carter, 2021). As the MD does not receive sensory or motor inputs, it is considered a high order nucleus. Instead, the MD receives inputs from the mPFC, thalamocortical neurons from other associative cortices, and several subcortical structures (Mitchell and Chakraborty, 2013; Ketz et al., 2015). Afferents from the mPFC emerges exclusively from layer VI of ACC, PL and IL (Gabbott et al., 2005). Lesion or inhibition of the MD leads to deficits similar to prefrontal lesions (Mitchell and Chakraborty, 2013; Parnaudeau et al., 2013), suggesting a close association between the MD and cognitive functions governed by the PFC.

The mPFC also receives dense projections from other associative cortical structures. One of the most studied connections of the mPFC is with the hippocampus (HPC) (Eichenbaum, 2017), structure involved in the representation of spatial and temporal sequences (Buzsáki and Tingley, 2018). Prefrontal-hippocampal interaction is relevant for object- and place-recognition memory (Chao et al., 2020, 2022), goal-directed spatial navigation and memory (Ito, 2018) and long-term consolidation of declarative memories (Girardeau and Zugaro, 2011; Euston et al., 2012). The HPC is subdivided into dorsal-HPC (dHPC) and ventral-HPC (vHPC). The dHPC is associated with cognitive operations, while the vHPC is mostly related with emotional and contextual-spatial processing (Lee et al., 2017). The vHPC is directly connected with the mPFC, in which excitatory neurons from the CA1 and the subiculum projects to the deep layers (V and VI) of the IL and PL (Hoover and Vertes, 2007; Anastasiades and Carter, 2021). Through this pathway, the vHPC may send contextual information to the mPFC (Cohen and Meyer, 2020). On the other hand, the dHPC is bidirectionally connected with the mPFC through the nucleus reuniens (RE) of the thalamus (Baker and Bird, 2002; Vertes, 2004; Joyce et al., 2022). The RE display bidirectional connectivity with the mPFC and the dHPC (Vertes et al., 2007). Simultaneously, the RE is the major thalamic input to the HPC, which distributes densely to CA1, the ventral subiculum, and entorhinal cortex (EC) (Jay and Witter, 1991; Vertes et al., 2007). This connectivity of the mPFC with the dHPC has been associated with the integration of spatial-temporal information (Eichenbaum, 2017).

The EC, cortical area that constitutes the hippocampal-entorhinal loop associated with spatial cognition and memory (Kitamura et al., 2015), send direct projections to the ACC and PL (Hoover and Vertes, 2007). These projections emerge from PN located in layer V and VI of the EC, innervating the superficial layers of the mPFC (Insausti et al., 1997). Given that the main output from the HPC is the EC, afferences from the EC may inform the mPFC about spatial-temporal features. On the other hand, the PL and IL project directly to the EC (Vertes, 2004). The posterior parietal cortex (PPC), area involved in the active guidance of the body through the visual space (Whitlock et al., 2008), projects densely to the ACC, but much less to the PL and IL (Kolb and Walkey, 1987; Vertes, 2004). This pathway may integrate information relative to self-motion into the mPFC (Whitlock et al., 2008). Direct projections from the ACC, PL and IL to PPC are scarce, although strong projection to the PPC emerges from the OFC (Olsen et al., 2019). The retrosplenial cortex (RSC) is another associative cortical structure connected with the mPFC. Although the specific function of the RSC has been difficult to clarify, it seems involved in the cross-modal integration during spatial navigation processing (Vann et al., 2009). The RSC is reciprocally connected with the ACC and PL (Jones et al., 2005; Hoover and Vertes, 2007). Interestingly, the RSC is also reciprocally connected with the EC and PPC, and receives unidirectional projections from the dHPC (Mitchell et al., 2018). Therefore, the RSC may participate in the integration of spatial and action-based information into the mPFC. The mPFC also receives restricted projections from the M2 cortex (Hoover and Vertes, 2007), area involved in the transformation of sensory cues into motor actions (Olson et al., 2020). The M2 is one main outputs from the mPFC, in which prefrontal afferences to M2 emerges from PL and ACC (Bedwell et al., 2014). Thus, M2 may participate in the organization of goal-directed motor actions.

Much of the cognitive control implemented by the mPFC is mediated through projections to several subcortical structures. Emotional control, for example, seems to be mediated by the connection between the mPFC with the amygdala (LeDoux, 2000; Marek et al., 2013). The amygdala is subdivided into the basal (BA), lateral (LA), and central nucleus (CeA) (LeDoux, 2000). The lateral and basal amygdala conform the basolateral complex of the amygdala (BLA), which is strongly innervated by the mPFC; these projections emerge preferably from layer II and V from ACC, PL and IL (Vertes, 2004; Gabbott et al., 2005). These prefrontal afferents to the amygdala may have a key role in goal-directed responses to threats (Alexandra Kredlow et al., 2022). Importantly, the BLA also send excitatory projections to the layer V of the PL and IL (Orozco-Cabal et al., 2006; Hoover and Vertes, 2007), whereas the CeA, considered the output of the amygdala, sends GABAergic afferent to the mPFC (Seo et al., 2016). These projection may integrate emotional (especially aversive) information into the mPFC. The nucleus accumbens (NAc), part of the mesolimbic dopaminergic reward circuitry (Floresco, 2015), is also strongly innervated by the mPFC (Vertes, 2004; Gabbott et al., 2005). Excitatory afferents to NAc emerges bilaterally from layer II, V and VI from PL and IL (Gabbott et al., 2005). Projections from the NAc to the mPFC seems to be absent (Hoover and Vertes, 2007). This mPFC-NAc circuitry may be relevant for guiding of behaviors according to rewards. The mPFC also projects to the lateral hypothalamus (LH), structure involved in the control of food intake and motivated behaviors (Stuber and Wise, 2016). The LH receive strong projections from layers II, III, V, and V from the ACC, PL and IL (Gabbott et al., 2005). This circuit participates in the cognitive control of food intake (Azevedo et al., 2022).

The mPFC also receive projections from several subcortical neuromodulatory nucleus. The dorsal raphe nucleus (DRN), the pedunculopontine tegmental nucleus (PPT), the locus coeruleus (LC), ventral-tegmental area (VTA) and the basal forebrain strongly project to the PL and IL (Hoover and Vertes, 2007; Henny and Jones, 2008). This monoaminergic and cholinergic innervation may modulate prefrontal network dynamics (Cools and Arnsten, 2022). Also the mPFC project to several of these nucleus. The DRN receive projections from layer V of the ACC, PL and IL (Gabbott et al., 2005), and the VTA receive projections from layer V of PL and IL (Gabbott et al., 2005). The mPFC also projects to the locus coeruleus (Cardenas et al., 2021) and the basal forebrain (Gaykema et al., 1991). This prefrontal innervation of monoaminergic and cholinergic nucleus may have a relevant role in global state of arousal (Mashour et al., 2022).

## 2 Neurophysiological basis for prefrontal cognitive control of behavior

### 2.1 Cognitive relevant features are represented by synchronized neuronal firing in the mPFC

How does the mPFC participate in the implementation of cognitive control? The most accepted hypothesis postulates that brain operations are supported by the transient, discrete, and strongly interconnected active ensembles of neurons, known as “neuronal assembly” (NA) (Hebb, 1949). This hypothesis proposes that NAs are made up of a relatively small set of distributed neurons that, by synchronized firing, encode relevant behavioral parameters (Buzsáki, 2010). Experimentally, a NA is a task-related synchronized overlapping firing of multiple single-neurons (Sakurai, 1999). Given that detection of NAs requires highly invasive intracerebral multielectrode recordings (Buzsáki, 2004) or fluorescent cell-imaging in behaving subjects (Carrillo-Reid et al., 2017), most studies relating NAs to cognitive features have been performed in animal models.

To date, the best characterized NA are the “place cells” in the HPC that encode the spatial position of the subject in the environment (Moser et al., 2008). Considering the role of the mPFC, NAs in this structure would represent multiple sets of discrete information reflecting cognitive-relevant elements, such as maintenance of information, strategies, decisions, and goals (Sakurai et al., 2013). Consequently, it has been found that prefrontal NAs encode a wide range of behavioral requirements for the task (Jung et al., 1998) including prospective goal choices (Baeg et al., 2003; Fujisawa et al., 2008; Benchenane et al., 2010), spatial goals (Hok et al., 2005) or strategy selection and switching (Rich and Shapiro, 2009; Powell and Redish, 2016). These NAs in the mPFC have been shown to be highly dynamical, as they are progressively formed in parallel with learning (Baeg et al., 2007; Benchenane et al., 2010); are transiently activated, reflecting the emergent dynamics of cognitive operations (Fujisawa et al., 2008), and once formed, can be activated at remote temporal scales, representing the long-term memory of the task (Baeg et al., 2007). Importantly, given their dynamic nature, prefrontal NAs support cognitive flexibility through abrupt changes in firing patterns related to the accommodation of new behavioral strategies as animals detect variations in their environment (Rich and Shapiro, 2009; Powell and Redish, 2016; Malagon-Vina et al., 2018). Thus, the formation, activation, and dynamic modulation of synchronized firing patterns of neuronal populations in the mPFC seem to be relevant for the implementation of cognitive control.

### 2.2 Oscillatory activity in the mPFC

Together with synchronized neuronal spiking, oscillatory patterns in the mPFC seems to support cognitive operations. Brain oscillations refer to rhythmic electrical activity detected as periodic fluctuations of the extracellular electric potential (i.e., local field potential, LFP) (Buzsáki et al., 2012; Yener and Başar, 2013). It reflects the non-linear summation of post-synaptic potentials that emerge from the synchronized interplay between excitatory and inhibitory synaptic transmembrane ion currents of the neural population (Pesaran et al., 2018). As brain oscillations are intrinsically periodic, they are classified into different bandwidths (between 0.5 and 200 Hz) that are related with different brain states (Buzsáki and Draguhn, 2004; Buzsáki and Watson, 2012). The amplitude and frequency of oscillations depends on the identity and neural composition of the neural network, the sum of the synchronized activity of these neurons, and the neuronal morphology and disposition of neurons in the cerebral space (Buzsáki and Draguhn, 2004; Maling and McIntyre, 2016). Thus, different oscillatory frequencies reflect the synchronized recruitment of different levels of neural populations, in which low-frequency oscillations are the manifestation of the synchronized activity of large-scale neural populations, whereas high-frequency oscillations represent the coordinated activity of local neural populations (Buzsáki and Draguhn, 2004; Rosanova et al., 2009).

Theta oscillation (6–12 Hz) is the most prominent low-frequency oscillatory activity observed in the mPFC. It is evident during locomotion and high cognitive demands (Colgin, 2011; Gordon, 2011). It is classically proposed that neocortical theta is driven by the HPC, which is thought to be sustained by inputs from the medial septum (cholinergic and GABAergic) and entorhinal cortex (GABAergic) (Buzsáki, 2002). However, hippocampal-independent theta has also been shown in the neocortex (Benchenane et al., 2011) although the exact circuit involved in its origin is still matter of discussion (Mitchell et al., 1982; Jeffery et al., 1995; Buzsáki, 2002; Schlesiger et al., 2015; Headley and Paré, 2017). During the last decade, a 4-Hz low-frequency oscillation has been described in the mPFC (Fujisawa and Buzsáki, 2011; Biskamp et al., 2017; Karalis and Sirota, 2022). Although the cellular origin of this rhythm is still unknown, it has been shown that it is locally generated in cortical networks by respiratory influence through the afferents from olfactory bulb (Folschweiller and Sauer, 2021). Given that this rhythm is also detected in several structures connected with the mPFC, as the HPC (Yanovsky et al., 2014), PPC (Jung et al., 2022), VTA (Fujisawa and Buzsáki, 2011), BLA (Karalis et al., 2016) and striatum (Oberto et al., 2022), this rhythm may synchronize distant structures with the mPFC. This 4-Hz oscillation is particularly evident in moments of immobility, suggesting that it may supports distributed coordination of neural networks when locomotion is absent (Biskamp et al., 2017). Evidence suggests that 4-Hz may be relevant for cognitive functions (Fujisawa and Buzsáki, 2011; Karalis et al., 2016; Bagur et al., 2021; Oberto et al., 2022). On the other hand, beta (15–30 Hz) and gamma (30–100 Hz) oscillations are the most prominent high-frequency oscillatory patterns in the mPFC. The origin of these interplayed oscillatory rhythms seems to depends in the interaction of local GABAergic and glutamatergic neurons (Bitzenhofer et al., 2017; Cardin, 2018). Even more, some studies link their origin to different subtypes of local GABAergic INs. For example, in the primary visual cortex of behaving mice, optogenetic differential stimulation of SST-IN or PV-IN are preferentially correlated with enhancement of beta or gamma activity, respectively (Chen et al., 2017). Although classical works provide support to the role of peri-somatic inhibition in gamma rhythmogenesis (Cardin et al., 2009; Sohal et al., 2009), it has also been shown that gamma can be driven by SST-IN, PNs or long-range GABAergic neurons projecting to the cortex (Adesnik and Scanziani, 2010; Kim et al., 2015; Veit et al., 2017). In summary, the neuronal composition (subtypes of neurons) and architecture (cell morphology and disposition of cells in space), as well as synchronized activity of cells are important factors needed for the emergence of oscillations at particular frequencies.

Astrocytes (a subtype of glial cell) also participate in the maintenance and modulation of brain rhythms (Buskila et al., 2019). Since they have a close association with synapses, they regulate the concentration of extracellular ions and neurotransmitters (tight control of the extracellular K+, glutamate uptake and gliotransmission). Also they communicate through calcium waves (astrocytic communication via gap junction), mechanisms by can influence brain activity and synchronization (Amzica et al., 2002; Lee et al., 2014; Bellot-Saez et al., 2018; Buskila et al., 2019). Indeed, astrocytic calcium dynamic is relevant for the modulation of hippocampal theta activity, and attenuation of IP3-mediated Ca^+2^ signaling in astrocytes increase theta power, especially during REM sleep (Foley et al., 2017). Higher frequency rhythms are also modulated by astrocytes; blockade of glutamate vesicular release from astrocytes induces a decrease in gamma power *in vitro* and *in vivo*, thus demonstrating their relevance for cortical gamma oscillations (Lee et al., 2014). Moreover, in a mouse model of astrocyte-specific exocytosis impairment (blockade of gliotransmitter release, presumably D-serine), the mPFC-HPC theta synchronization was impaired, as well as cognitive tasks associated with spatial learning and reference memory (Sardinha et al., 2017). Thus, these result evidence the modulation of network dynamics by astrocytes and their impact in functional communication (Sardinha et al., 2017).

Together with cell composition and the architecture of neural networks, the genetic background of cells is relevant for the generation of brain rhythms (Buzsáki et al., 2013). Indeed, it has been shown that brain oscillations are highly heritable (Peeters et al., 1992; van Beijsterveldt et al., 1996; Franken et al., 1998; Buzsáki et al., 2013; Müller et al., 2017). In humans, EEG profiles show higher similarity between monozygotic twins compared with between dizygotic twins or unrelated people (Landolt, 2011). Additionally, genetic mutations can generate disease states; for example deletions or duplications of the SCN1A gene cause Dravet syndrome; these patients show an impaired ability to generate gamma activity in response to auditory stimuli compared to healthy controls (Sanchez-Carpintero et al., 2020). Moreover, current research has identified genes in the neocortex correlated with oscillatory activity linked to successful memory encoding (Berto et al., 2018, 2021). These genes are expressed mainly in neurons, which encode ion channels and synaptic proteins (Berto et al., 2018, 2021). In a recent work performed in humans, it was observed that genes encoding for ion channel activity, chromatin remodeling, synaptic scaffolding, and alternative splicing were related to successful memory encoding (Berto et al., 2021; Khanna and Williams, 2021). Future research will clarify the mechanisms of genetic control of brain oscillations.

A key feature of cortical brain oscillations is that they are internally generated, even in the absence of external cues; hence, brain oscillations may represent internal-generated neural operations (Buzsáki et al., 2014). Thus, particular oscillatory patterns may emerge in the mPFC according to cognitive requirements, representing relevant features related to diverse cognitive and behavioral tasks (Hyman et al., 2005; Benchenane et al., 2010; Buzsáki, 2010; O’Neill et al., 2013). For example, theta oscillations appear in the rodent mPFC largely associated with the performance of spatial tasks (Siapas et al., 2005; O’Neill et al., 2013), and learning and memory consolidation (Benchenane et al., 2010; Alekseichuk et al., 2016). This prefrontal theta is usually coupled with hippocampal theta (see below) (Hyman et al., 2005; Jones and Wilson, 2005; O’Neill et al., 2013). On the other hand, high-frequency oscillations also emerges in the mPFC, which may represent local neural operations underlying information processing (Fries, 2009; Engel and Fries, 2010; Fernandez-Ruiz et al., 2023). For example, synchronization of mPFC with other cortical areas in the beta band in primates, humans and rodents is associated to top-down attention (i.e., expectation based attention), while synchronization in gamma prevails during bottom-up attention (i.e., states more focused in features of the presented stimuli) (Buschman and Miller, 2007). Similarly, during cognitive flexibility, increases in gamma activity (anterior cingulate and right temporo-parietal cortex) and decreases in alpha and beta (frontal and inferior-parietal cortex), have been related to improved performance during task-switching paradigms in humans (Proskovec et al., 2019). Cortical low-frequency rhythms, as theta oscillations, modulate the timing and amplitude of high-frequency rhythms, a phenomenon known as cross-frequency coupling (CFC) (Canolty and Knight, 2010; Lisman and Jensen, 2013; Aru et al., 2015). This phase-to-amplitude modulation allows the coordination of fast-local computations by slower oscillations at larger spatial scales, offering windows of efficient communication between different neural networks, allowing the integration of distributed local computations into large-scale processes (Canolty and Knight, 2010). This theta-gamma CFC modulation has been evidenced in the mPFC during the performance of several cognitive functions in rodents (Fujisawa and Buzsáki, 2011; Li et al., 2012; Tamura et al., 2017) and non-human and human primates (Voloh et al., 2015; Daume et al., 2017; Jones et al., 2020). Also 4-Hz rhythm is capable of synchronizing gamma oscillation in the mPFC, although its role on cognitive operations is still under research (Fujisawa and Buzsáki, 2011; Zhong et al., 2017; Karalis and Sirota, 2022). Interestingly, spiking neurons in the mPFC are also entrained by theta-gamma CFC (Fujisawa and Buzsáki, 2011; Li et al., 2012; Tamura et al., 2017) which has been hypothesized as a mechanism for integration and segregation of task-relevant neural populations. Thus, CFC seems to be critical for cognitive control (Helfrich and Knight, 2016).

Importantly, oscillations modulate the timing of the membrane potential of recruited neurons, synchronizing the timing of action potentials (Anastassiou et al., 2011). Thus, the synchronization of neural firing and oscillatory rhythms are not independent phenomena; contrarily, the synchronization of neuronal firing by oscillations seems to support the formation and activation of NAs through the integration of activity-dependent synaptic plasticity (Fell and Axmacher, 2011; Buzsáki and Watson, 2012). Therefore, oscillatory synchronization of neuronal spiking seems to be a requisite for the formation of NAs (Buzsáki, 2010). As an example, the timing and sequence of place cells in the HPC are synchronized by theta and gamma oscillations (Buzsáki and Moser, 2013). The capability of brain oscillations to modulate the timing and occurrence of neuronal spiking activity is commonly measured through phase-locking (Lowet et al., 2016). It has been shown that prefrontal oscillations, throughout phase-locking, synchronized prefrontal spiking that encode relevant event-related information for the behavioral task, promoting the generation and activation of cognitive relevant NAs (Benchenane et al., 2010; Negrón-Oyarzo et al., 2018). Thus, cognitive control of behavior may be supported by the coupling of NAs by different and complementary patterns of oscillatory activity in the mPFC.

### 2.3 Functional connectivity between the mPFC and distributed neural networks for the cognitive control of behavior

For the implementation of cognitive control, the mPFC requires rapid and flexible information exchange among anatomically connected structures that represents and store relevant information required for task performance (Euston et al., 2012; Buzsáki et al., 2014; Helfrich and Knight, 2016). It has been proposed the large-scale synchronization of activity patterns, known as functional connectivity (FC), is a neural mechanism for neural communication, allowing the integration of local computations across different spatio-temporal scales (Fries, 2005; Friston, 2011; Eickhoff and Müller, 2015). Consequently, FC between the mPFC and distributed neural networks may facilitate the dynamic integration and coupling of information crucial for the formation and activation of NAs involved in cognitive control (Colgin, 2011; Helfrich and Knight, 2016).

The mPFC shows strong FC with the HPC. FC is commonly measured as spectral coherence defined as the cross-correlation of both amplitude and phase as a function of frequency and time between two LFP signals (Gordon, 2011). Coherence in the theta frequency between the mPFC and HPC has been widely described, which increases in relationship with cognitive performance, such as decision making, spatial memory acquisition and inhibitory control (Adhikari et al., 2010b; Benchenane et al., 2010; O’Neill et al., 2013; Negrón-Oyarzo et al., 2018). Interestingly, this interaction modulates the spike timing of prefrontal neurons (Jones and Wilson, 2005; Siapas et al., 2005; Benchenane et al., 2010; Negrón-Oyarzo et al., 2018), allowing the emergence of neural spiking representations of relevant cognitive features during learning (Adhikari et al., 2010a,b; Benchenane et al., 2010). Also, there is a prominent coherence in the low-gamma band (20–40 Hz), which progressively increases over time through task acquisition, suggesting that the FC between mPFC-HPC supports strategy progression during learning (Negrón-Oyarzo et al., 2018). Hippocampal theta oscillation also coordinates prefrontal gamma oscillations though CFC (Sirota et al., 2008). This phenomenon has been associated with working memory (Fujisawa and Buzsáki, 2011; Li et al., 2012; Tamura et al., 2017). Given that gamma oscillations represent local computations (Fries, 2009; Fernandez-Ruiz et al., 2023) this long-range synchronization may allow the coordination and integration of distributed computations, favoring neural communication and plasticity required for cognitive control (Hyafil et al., 2015; Helfrich and Knight, 2016). The mPFC-HPC coupling may be relevant for the integration of spatial and temporal information into the mPFC required for cognitive control. However, it has been shown that the directionality of mPFC-HPC coupling may represents different processes: for example, HPC-to-mPFC participates in the transference of contextual information to the mPFC, whereas mPFC-to-HPC coupling guides successful retrieval of memories in the HPC (Place et al., 2016). Therefore, mPFC-HPC coupling may also represent cognitive control exerted by the mPFC. The coordination between the mPFC and HPC is also attained by 4-Hz oscillation (Karalis and Sirota, 2022). Similarly to theta, 4-Hz oscillation also coordinates gamma activity and neuronal spiking at long-range. However, contrary to theta coordination, 4-Hz synchronization emerges during offline states, when locomotion is not present (Karalis and Sirota, 2022). This coordination may be a complementary mechanism for neural communication when theta is absent (Folschweiller and Sauer, 2021).

As an accumulative process, the acquisition of goal-directed memory requires access to previous experiences stored in long-term memory and the formation of new long-term memories through memory consolidation (Mecklinger, 2010). The mPFC-HPC axis plays a pivotal role in supporting memory consolidation (Rothschild et al., 2017; Shin et al., 2019; Wagner et al., 2019). The most accepted current model form memory consolidation is the “two-stage model” (Buzsaki, 1989) that proposes an online stage, where environmental information is acquired, and an offline stage, where recently acquired information is transferred to distributed cortical modules for long-term storage (Nieuwenhuis and Takashima, 2010; Preston and Eichenbaum, 2013). During spatial memory formation, the online stage is characterized by a peak of coherence in theta between the HPC and mPFC, facilitating the coordination of neuronal firing in the mPFC and the formation of NAs to store relevant task-related information (Siapas et al., 2005; Sirota et al., 2008; Benchenane et al., 2010; Wang et al., 2020). Theta oscillations are believed to play a crucial role in “tagging” NAs for later consolidation (Peyrache et al., 2009; Girardeau and Zugaro, 2011; Jadhav et al., 2016). The offline stage, occurring during sleep or quiet wakefulness, involves reduced external stimulation (Wamsley, 2019). This stage is associated with the interaction of three major structures: the mPFC through slow oscillations (SO: <1 Hz), the thalamo-cortical circuit through spindles (10–16 Hz), and the HPC through sharp-wave ripples (SWR: 120–250 Hz) (Binder et al., 2019; Oyanedel et al., 2020). SWR in the HPC leads to the reactivation of hippocampal sequences formed during the online stage (Peyrache et al., 2009). Importantly, prefrontal neurons are reactivated during SWR, indicating the interaction between the mPFC and HPC during the consolidation process (Jadhav et al., 2012; Rothschild et al., 2017; Tang et al., 2017). This hippocampal reactivation during the offline stage allows the transfer of relevant information acquired during the online stage, contributing to the strengthening of synaptic connections and the consolidation of acquired information in the long-term (Frankland and Bontempi, 2005; Papale et al., 2016; Binder et al., 2019). Inhibition of SWR during the offline stage impairs the acquisition of goal-directed spatial memory, reinforcing the crucial role of the mPFC-HPC axis in the memory consolidation process (Girardeau et al., 2009; Jadhav et al., 2012; Binder et al., 2019). This evidence suggests the relevance of the mPFC-HPC coupling for several processes related to cognitive control.

It also has been documented FC between the mPFC and MD. For example, it has been observed coherence at beta frequency in the mPFC-MD circuit during working memory and decision-making (Parnaudeau et al., 2013; Bolkan et al., 2017). Specifically, in working memory tasks, it has been found that MD is related with the “online holding” of relevant information, whereas the mPFC is related with the execution of actions (Bolkan et al., 2017). The MD also may play a role in memory consolidation (Mitchell and Gaffan, 2008; Cross et al., 2012). Given that the MD decrease their firing rate when SWR emerges in the HPC (Logothetis et al., 2012; Yang et al., 2019), it has been suggested that that MD contributes to increase the mPFC reactivity to hippocampal SWR. This mechanisms may promote hippocampal-cortical communication for the consolidation of declarative memory (Yang et al., 2019).

FC between the mPFC and the amygdala may contribute to cognitive control of emotional processing (Alexandra Kredlow et al., 2022). Most of studies concerning mPFC-amygdala coupling have been performed using fear conditioning and extinction task. Early studies showed that theta oscillation is evident in the rodent LA during retrieval of fear memory (Pape et al., 2005). Interestingly, theta coherence between BLA and mPFC predict freezing (Popa et al., 2010) and successful fear discrimination (Likhtik et al., 2014). This has also been observed in human and non-human primates (Taub et al., 2018; Chen et al., 2021). However, as well as the mPFC-HPC interaction, the directionality of mPFC-amygdala coupling may signal different cognitive processes. For example, BLA-to-mPFC coordination has been associated with communication of aversiveness to the mPFC (Popa et al., 2010; Taub et al., 2018), whereas mPFC-to-BLA modulation was associated with prefrontal control of fear expression (Popa et al., 2010; Courtin et al., 2014) and successful threat evaluation (Likhtik et al., 2014). Interestingly, it has been shown that the directionality of mPFC-amygdala coordination in the theta band depends on fear responses during different stages of fear memory and extinction (Lesting et al., 2013). This suggests that long-range communication between the mPFC and the amygdala through theta oscillations depend on the current cognitive process. The mPFC and amygdala also interact through of theta-gamma CFC, which increase in periods of fear, and is differentially modulated by task requirements (Stujenske et al., 2014). 4-Hz oscillation also seems to have a central role in emotional processing in the mPFC-amygdala circuit (Folschweiller and Sauer, 2021). 4-Hz synchronization between mPFC and BLA increase during freezing behavior, in which mPFC-4-Hz entrain BLA oscillations and neural spiking, suggesting a role in top-down control of fear expression (Dejean et al., 2016; Karalis et al., 2016). Importantly, both theta and 4-Hz oscillations synchronizes neuronal spiking between the mPFC and amygdala, contributing to the formation of NAs signaling specific parameters related to the task, as fear expression or extinction (Courtin et al., 2014; Likhtik et al., 2014; Dejean et al., 2016; Karalis et al., 2016). Altogether, this body of evidence strongly suggests that functional connectivity between the mPFC and anatomically connected structures supports several features of cognitive control of behavior.

## 3 Dysfunctional activity patterns in the mPFC in mental conditions

**FIGURE 1:**
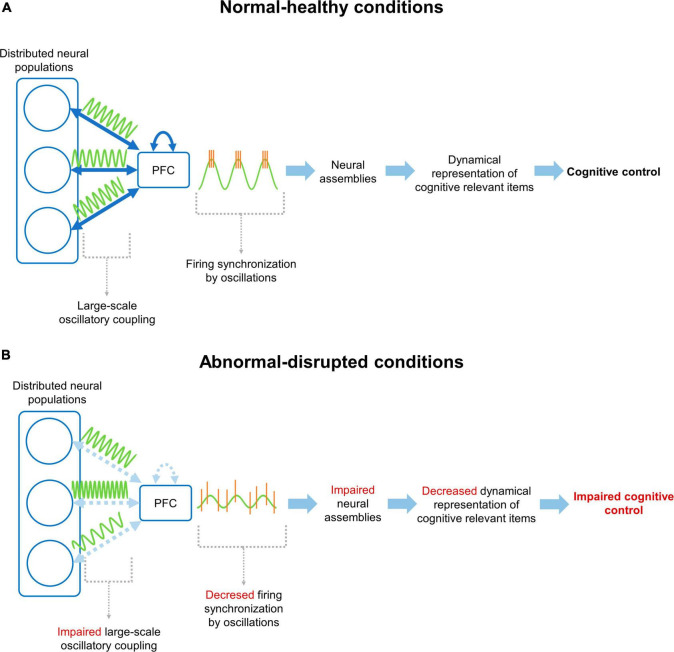
Neural mechanism for cognitive control through prefrontal cortex functional connectivity. **(A)** Under normal/healthy conditions, the PFC communicates with distributed neural populations through large-scale coupling of oscillatory activity patterns (i.e., functional connectivity). This coupling facilitates the synchronization of firing patterns in the PFC, promoting the formation, activation and updating of cognitively relevant NAs, supporting the cognitive control of behavior according to current demands. **(B)** On the other hand, under disrupted conditions, the connectivity between the PFC and distributed networks, or between prefrontal neurons, is altered. This prevents coupling between the mPFC and distributed networks, decreasing the synchronization of neuronal firing and hindering the organization of NAs. Finally, all this chain of events manifests as an impaired cognitive control of behavior.

The evidence presented above links cognitive control with neural activity patterns in the mPFC and its functional connectivity with distributed networks (Figure 1A). Considering that some normal and pathological conditions display a strong alteration in the cognitive control of behavior, it is expected that neurophysiological processes supporting this operation would be also impaired. However, the limited access to the recording and modulation of neural activity patterns in human subjects restrains the knowledge relating neurophysiological phenomena to the decline of cognitive control. Therefore, studies in animal models have been proposed and used to address this issue. Given the development of reliable rodent models mimicking normal and pathological mental conditions in humans, we can assume that the decline in cognitive control in these rodent models can be similar to that in humans. Thus, the integration and comparison between finding in humans and rodent models could give relevant cues to the understanding of neurophysiological mechanisms involved in decline of cognitive control in normal and pathological conditions. In the following sections we discuss how prefrontal activity patterns and cognitive control are altered in patients and rodent models of normal cognitive aging, mood disorders and schizophrenia.

### 3.1 Prefrontal oscillations and functional connectivity in normal cognitive aging

Aging has become a relevant topic in research because of the global increase in the proportion of older people. According to the World Health Organization (WHO), people aged 60 years or older currently reaches near 12% of the global population. Furthermore, it is projected that in the year 2050, the world population of people aged 60 years or older will have doubled (2.1 billion of people), reaching 22% of the total population (World Health Organization [WHO], 2022). Aging is a natural and irreversible process characterized by biological and social changes (Dziechciaż and Filip, 2014). However, elderly are also accompanied by a normal cognitive decline (NCA), a natural and gradual decline in cognitive functions that occurs normally over time, which differs from pathological syndromes, such as mild cognitive impairment and dementia (Harada et al., 2013; Dumas, 2015). NCA start at 40 years of age and progresses continuously (Harada et al., 2013). It has been estimated that between 3 and 8% of the population older than 65 years of age show a level of cognitive decline, whereas this proportion rise to 30% in the population older than 85 years (Blazer et al., 2015). This phenomenon strongly impact the autonomy and well-being of older people (Sánchez-García et al., 2019; Cylus and Al Tayara, 2021).

NCA is characterized by a decrease in “fluid” cognitive abilities, such as cognitive flexibility, problem-solving, and reasoning (Manard et al., 2014). In contrast, crystallized abilities, as accumulated knowledge of the world and over-learned familiar skills remain intact (McDonough et al., 2016; Salthouse, 2019). This differs from pathological conditions like dementia, in which more widespread brain functions impairment is observed, affecting both fluid and crystallized abilities, leading to a broader decline in cognitive capacities (Cadar, 2018). Deterioration of cognitive control is one of the main characteristics of NCA (Paxton et al., 2008), manifested as a diminished capacity to process new information and adapt to changing situations (Harada et al., 2013). Indeed, the cognitive decline is manifested as a deterioration of executive functions including working memory and cognitive flexibility (Paxton et al., 2008; Reinhart and Nguyen, 2019; Yagi et al., 2020). Notably relevant is the impairment of spatial memory (Head and Isom, 2010; Gazova et al., 2013; Bécu et al., 2023). Although these deficits are related to spatial components, as for example, spatial orientation (Moffat et al., 2006; Bécu et al., 2023), impairment on executive functions, such as strategy switching or attention, are significantly associated with deficits of spatial memory during aging (Rodgers et al., 2012; Wiener et al., 2013; Harris and Wolbers, 2014; Zhong and Moffat, 2018). This evidence suggests a relationship between cognitive decline and prefrontal function (West, 1996; Greenwood, 2000).

During aging, there is a general reduction in cortical thickness, volume, and weight of the brain (Dekaban and Sadowsky, 1978; Takao et al., 2012; Zheng et al., 2019; Cox et al., 2021). However, the reduction of cortical thickness is especially evident in the PFC (Dotson et al., 2015). Importantly, intrinsic and long-range anatomical connectivity of the PFC with distributed structures is decreased (Chadick et al., 2014; Pietrasik et al., 2023), which is associated with cognitive performance (Chadick et al., 2014). At neurophysiological level, EEG/MEG studies in humans have documented a decrease in the power of frontal low-frequency oscillations in aged subjects. For example, average power spectral density of theta frequencies is reduced in older subjects compared to young individuals (Vlahou et al., 2014; Meghdadi et al., 2021). Similarly, subjects with NCA display a decrease in spectral power at frequencies below 14 Hz in frontal superior and inferior areas during word-memory tasks (Healey and Kahana, 2020). In the same line, it has been shown that there is a decrease in theta (4–7 Hz), and alpha (9–14 Hz) power during memory tasks in the frontal areas of NCA patients compared to young adults (Rondina et al., 2016). During the resting state, the spectral power density of theta frequencies was significantly correlated with immediate and delayed verbal recall, attention, and executive function measures in older adults (Cummins and Finnigan, 2007; Finnigan and Robertson, 2011). Similarly, decrease of alpha rhythms in the frontal area correlates with performance during working memory task in older subjects (Clark et al., 2004). Interestingly, the evidence suggests that cognitive decline in working memory and behavioral flexibility in old age is associated with the difficulty of the PFC to synchronize at large-scale with distributed brain regions, such as temporal lobe and thalamus (Fama and Sullivan, 2015; Hakun et al., 2015; Reinhart and Nguyen, 2019). This evidence suggests that during the requirement of high cognitive demand, the cognitive decline in aging appears to be associated with the impairment of the slow-frequency synchronization of neural networks in the PFC, which could be related to the inability to recruit neural circuits during the task.

Naturally aged rodents (18–24 months) is the most common model for the study of aging (Yanai and Endo, 2021). Similar cognitive impairments to those observed in NCA in humans have been found in these rodent models (Brito et al., 2023). For instance, aged rodents display impairments in spatial memory (Rapp et al., 1987; Gallagher, 1997; Drapeau et al., 2003; Magnusson et al., 2003; Guidi et al., 2015; Lester et al., 2017), working memory, and strategy switching (Barnes et al., 1980; Breton et al., 2015; Yanai and Endo, 2021; Chong et al., 2023). Despite the existence of rodent models of aging, there are scarce studies concerning the neurophysiological phenomena in the mPFC related to the decline of cognitive control during aging. As in humans, aging in rodents is associated to a reduction in cortical thickness, volume, and weight of the rodent brain (Lessard-Beaudoin et al., 2015; Taylor et al., 2020). At neurophysiological level, and in agreement with human data, aged C57BL/6J mice show low power of theta and high-frequency oscillations during resting state in the mPFC (Rumschlag et al., 2021). Studies assessing the neuronal activity patterns in the mPFC related to impaired cognitive control during elderly is even scarcer. A recent study showed a decrease in the fraction of action-plan coding neurons in the mPFC of aged animals, which was related to slower learning in a working memory task (Chong et al., 2023). It has also been shown a reduced neural spike encoding of response latencies to stimuli in the mPFC during the delay period in an operant delayed-response task which was related with impaired performance (Caetano et al., 2012). These studies suggest a relationship between impaired neural encoding in the mPFC with the decline of cognitive abilities during aging. Altogether, these findings reflect that brain aging involves complex changes affecting prefrontal activity patterns, which may be key to understand the difficulties in the cognitive control observed during normal aging. Interestingly, brain stimulation mimicking theta-gamma CFC recovered working memory in older adults, supporting the role of prefrontal activity patterns in cognitive decline (Reinhart and Nguyen, 2019). Therefore, optogenetic stimulation, which offers high cell-identity, spatial and temporal precision, could be used in the mPFC of rodent models of aging, contributing to the development of evidence-based strategies to improve cognitive control in aged subjects.

### 3.2 Prefrontal oscillations and functional connectivity in mood disorders

Mood disorders includes a group of psychiatric diseases that affect the individual’s emotional processing, energy, and motivation. Some examples of these diseases are major depressive disorder (MDD) and anxiety disorders (AD). MDD has a lifetime prevalence of 16%, whereas anxiety disorders are even more prevalent than MDD, reaching up to 60% (Kessler et al., 2003). Genetic studies have shown a heritability of 37% for the generation of mood disorders. It also exists high comorbidity between both mental illnesses (Solomon et al., 2000). A large body of evidence indicates that subjects at higher risk of developing mood disorders are those who are genetically predisposed (Jaworska-Andryszewska and Rybakowski, 2019) and exposed to threatening and chronic life conditions, including people living in poverty, female victims of violence, the unemployed, neglected elderly persons and individuals exposed to bullying (McEwen and Akil, 2020). These threatening conditions generate a physiological response that allows adaptation to environmental threats, known as stress (McEwen, 2007). Stress-related mental diseases are characterized by a plethora of symptoms (American Psychiatric Association, 2013). However, the most important and invalidating features of mood disorders are related to cognitive control, which are strongly related to the impaired well-being of the patients. In this context, symptoms such as cognitive impairment, incapacity to control impulses and emotions are among the most relevant (Nezlek et al., 1994; Taylor Tavares et al., 2007; Hammar and Årdal, 2009; Millan et al., 2012). Current evidence shows that the PFC’s functioning is altered in mood disorders (Johnna and Swartz, 2013; Myers-schulz and Koenigs, 2014; Marrus et al., 2015; Mehta et al., 2018). One of the main components in mood disorders is the emotional dysregulation, i.e., the incapacity to regulate negative emotions. One study in humans showed that patients with MDD treated with antidepressants for 6 months presented an accelerated increase in the right dorso-lateral PFC activity during the regulation of negative affect in comparison to controls (Heller et al., 2013). Untreated MDD patients showed lower PFC activity in general, which altered the connectivity with the amygdala (Johnstone et al., 2007). This could be responsible for the characteristic emotional dysregulation symptom in this disease because this circuit is important for emotional processing. Changes in PFC engagement when regulating negative affect are inversely correlated with changes in depression severity (Johnstone et al., 2007). For social AD, it has been showed that patients presented lower amplitude fluctuations of low-frequency oscillations in the PFC in comparison to control patients (Zhang et al., 2015). Theta-gamma CFC alterations in the PFC is related to cognitive performance in MDD (Zheng and Zhang, 2013). Thus, this evidence supports the impairment of activity patterns in the PFC in mood disorders.

Several rodent models of mood disorders have been developed. Given the close relationship between mood disorders and chronic stress (McEwen and Akil, 2020), most of mood disorders models implicate the exposition of the animals to chronic-stressing conditions, such as chronic- and unpredictable stress, social-defeat stress, prenatal and early-life stress, or corticosterone manipulation, among others (Gururajan et al., 2019). These stressing conditions induce several behavioral manifestation of mood disorders, as increased anxiety, decreased locomotion and motivation, anhedonia, and social avoidance (Krishnan and Nestler, 2011; Wang et al., 2017; Gururajan et al., 2019). Also, these protocols induce cognitive impairment in spatial and recognition memory (Kleen et al., 2006; Conrad, 2010; Darcet et al., 2014), decision-making (Friedman et al., 2017), fear extinction (Negrón-Oyarzo et al., 2014) and behavioral flexibility (Hurtubise and Howland, 2017). Interestingly, during spatial learning, chronic stress induced a shift to more rigid stimulus-response strategies (Schwabe et al., 2008). This evidence suggests a stress-induced impairment in cognitive control.

It is widely documented that chronic stress induce dendritic pruning of PN in the mPFC (Cook and Wellman, 2004; Dias-Ferreira et al., 2009; Garrett and Wellman, 2009). At neurophysiological level, chronic stress reduced synaptic transmission in the mPFC (Yuen et al., 2012; Negrón-Oyarzo et al., 2014, 2015a) and reduce firing of prefrontal neurons (Mizoguchi et al., 2000; Goldwater et al., 2009; Wilber et al., 2011; Negrón-Oyarzo et al., 2015b). Similarly, social-defeat stress reduces spiking of prefrontal neurons (Abe et al., 2019). Thus, stress-related rodent models of mood disorders display profound alterations in prefrontal structure and function, which may be related to the impaired control of behavior. These neurophysiological impairments may impact in prefrontal activity patterns and FC during behavior implementation. Accordingly, it has been shown that chronic stress decreases mPFC-HPC coherence in the theta frequency band (Lee et al., 2011). Similarly, chronic stress reduced mPFC-HPC coherence at delta, theta and gamma bands during spatial memory task, which was related to the impairment of memory acquisition (Oliveira et al., 2013). Prenatal stress induced persistence of spatial memory during adulthood, suggesting a loss of behavioral flexibility, which was related to an increased synchronization between hippocampal-SWR and neuronal firing in the mPFC (Negrón-Oyarzo et al., 2015b). In a similar line, social-defeat stress induced a decrease in the 2–7 Hz oscillations in the mPFC, which correlated with stress-induced behavioral state (Kumar et al., 2014; Liu et al., 2022). Also, social-defeat stress reduced the incidence of 20–40 Hz events in susceptible animals during social interaction (Abe et al., 2019). Of relevance, social-defeat stress also reduced the prefrontal synchronization of neural spiking in the amygdala in susceptible animals (Kumar et al., 2014). If prefrontal neural representation and encoding of relevant cognitive evens is affected in rodent models of mood disorders is still unknown.

Finally, considering those antecedents, many prefrontal stimulation therapies have been developed to decrease symptoms of mood disorders (Cirillo et al., 2017). The search for new therapies is motivated by the limited effectiveness of pharmaceutical treatments (Nakagawa et al., 2017), as a great number of patients (between 12 and 55%, depending on the psychiatric illness) do not respond to this type of treatment (Nemeroff, 2007; Wiles et al., 2014). Stimulation therapies like deep brain stimulation (DBS) or transcranial magnetic stimulation (TMS) have been developed (Zrenner et al., 2020). Thus, precise, and evidence-oriented stimulation therapies may help patients to recover their abnormal oscillatory coupling characteristic of the pathophysiology of mood disorders. Therefore, rodent models of mood disorders are a reliable tool to test brain stimulation protocols (Okonogi and Sasaki, 2021). In rodent models, it has been shown that deep brain stimulation in the mPFC increased the synchronization between the HPC and the mPFC in the beta and gamma band, which may have an antidepressant-like effect, decreasing symptoms such as dysregulation in emotional processing (Jia et al., 2019). These amplitude fluctuations were related to the symptoms of the impairment. Interestingly, optogenetic stimulation of PN in the mPFC induced antidepressant-like effects in socially stressed mice (Covington et al., 2010; Kumar et al., 2013; Carlson et al., 2017). Similarly, optogenetic stimulation of prefrontal PN that project to DRN to amygdala recovered depressive-like behavior evaluated in the forced-swim test and social-interaction tests (Warden et al., 2012; Challis et al., 2014; Vialou et al., 2014). Of relevance, stimulation of prefrontal afferents to striatum recovered decision-making deficits induced by chronic stress (Friedman et al., 2017), suggesting that specific brain stimulation in the mPFC may recover cognitive control impaired in chronic-stress models of mood disorders (Biselli et al., 2021).

### 3.3 Prefrontal oscillations and functional connectivity in schizophrenia

Schizophrenia (SZ) is a severe mental disorder characterized by aberrant thoughts and behaviors. According to the WHO, it affects nearly 1% of the global population, reaching 21 million people worldwide (World Health Organization, 2012). SZ is most frequent in males, and the initial manifestation of the disease commonly appears in early adulthood (Häfner and an der Heiden, 1997). According to DSM-V (American Psychiatric Association, 2013) SZ is characterized by three main symptomatologic features: positive symptoms, which include delusion, paranoia and hallucinations; negative symptoms, such as abulia, alogia, anhedonia and avolition; and cognitive symptoms, manifested as an impairment on working memory, set-shifting, long-term memory recall, and selective attention (Goldman-Rakic, 1994; Hepp et al., 1996; Gold et al., 1997; Gallinat et al., 2004; Senkowski and Gallinat, 2015). These cognitive symptoms can be categorized as a detriment of executive control, in which the impairment in several forms of cognitive control have been considered as one of the most consistent, leading to the emergence of perseverative behaviors (Ridley, 1994; Crider, 1997; Lanser et al., 2002; Waltz, 2017). Thus, the deficit of cognitive control appears to be a hallmark of schizophrenia (Lesh et al., 2011).

Early neuroimaging investigations reported a decrease in the activation of the PFC in SZ patients (Ingvar and Franzén, 1974; Andreasen et al., 1992). Several of these findings have been largely replicated during the last decades (Shenton et al., 2001; Pomarol-Clotet et al., 2010; Penner et al., 2016). Importantly, reduced PFC activation has been correlated with impaired prefrontal-dependent cognitive operations in SZ (Perlstein et al., 2001, 2003). Thus, cognitive dysfunction observed in SZ is consistent with a deterioration of prefrontal function (Smucny et al., 2022). Structural connectivity analysis in SZ patients have revealed specific reduction of connectivity within the PFC and between the PFC with distributed structures (Kubicki et al., 2007). Prefrontal intrinsic connectivity is correlated with negative symptom (Hoptman et al., 2002; Wolkin et al., 2003) and decreased frontal-temporal connectivity is correlated with impairments in executive functions and memory (Kubicki et al., 2002, 2003). At a cellular level, postmortem studies revealed cytoarchitectonic alterations in the PFC of SZ, such as fewer dendritic spines in PN (Garey et al., 1998; Glantz and Lewis, 2000) reduction of neuropil (Selemon et al., 2004) and reduction of the mean clustering distance between cells (Casanova et al., 2008). Given its functional relevance, one of the most important post-mortem findings has been the reduction of the density of IN (Benes and Berretta, 2001) and GAD67 expressing cells in the PFC of SZ patients (Guidotti et al., 2000; Volk et al., 2000). Similar findings have been consistently found in several following postmortem studies (Beasley and Reynolds, 1997; Hashimoto et al., 2003, 2008; Bristow et al., 2015). Considering the role of INs in the emergence of brain oscillations, is not surprising that SZ patients displays aberrant oscillatory activity in the PFC (Uhlhaas and Singer, 2010; Senkowski and Gallinat, 2015; Hunt et al., 2017). Initial evidence reported an impairment in gamma oscillations. Specifically, it was found that the amplitude of gamma oscillations increased in the PFC of healthy controls when subjected to cognitive demands, effect not observed in SZ patients, which correlated with cognitive performance (Haig et al., 2000; Gallinat et al., 2004; Cho et al., 2006; Basar-Eroglu et al., 2007). This effect is observed in first episode SZ patients and is independent of medication status (Minzenberg et al., 2010). Importantly, the prefrontal cognitive-related impairment of gamma oscillations is the most consistent neurophysiological finding reported in SZ (Uhlhaas and Singer, 2010, 2013). This impairment of gamma is also observed at large-scale synchronization. For example, gamma synchronization between PFC and visual cortex is decreased in SZ patients, which correlated with the clinical state (Hirvonen et al., 2017). Interestingly, it has been found an increase of gamma oscillations during resting state in SZ patients (Boyden et al., 2005; Rutter et al., 2009; Kikuchi et al., 2011; Spencer, 2011; Andreou et al., 2015b; Grent-’t-Jong et al., 2018). This evidence suggests a difficulty to engage prefrontal gamma oscillations in SZ patients when it is required for cognitive control. Additionally, recent work has also found disturbances in theta oscillations in SZ patients. Similarly as for gamma oscillation, the impairment is evidenced as a decrease of prefrontal theta when the subject face cognitive challenge, such as working memory (Schmiedt et al., 2005; Griesmayr et al., 2014). Contrarily, it is observed an increased theta during resting state (Andreou et al., 2015a; Di Lorenzo et al., 2015). This impaired theta is also observed in long-range prefrontal coupling with distributed structures (Adams et al., 2020). In the same line, the increase of prefrontal theta-gamma- and delta-gamma CFC correlated with cognitive performance in healthy controls is not observed in SZ patients (Griesmayr et al., 2014; Missonnier et al., 2020). Therefore, the accumulated evidence suggests an impairment of gamma and theta oscillations, and in the interaction between these oscillation, in the PFC of SZ patients.

To date, several postulates have been developed to explain the neuropathology of SZ, as dopaminergic dysfunction (Knable and Weinberger, 1997), dysregulation of the excitatory/inhibitory balance (Lewis et al., 2005), and impaired cortical neurodevelopment (Garey, 2010). The combination of these hypotheses into an integrative vulnerability-stress model (known as a “two-hit model”), which combines genetic with pre- and postnatal environmental factors, has become one of the most accepted postulates for the development of SZ, which also reveals the complexity and interaction of several factors in its physiopathology (Bayer et al., 1999; Davis et al., 2016). Thus, considering these hypotheses, together with the most relevant factors for the development of SZ, several rodent models have been developed (Table 1), in which cognitive impairments are usually evident (Lipska and Weinberger, 2000; Marcotte et al., 2001; Jones et al., 2011; Winship et al., 2019; Speers and Bilkey, 2021). For example, neurodevelopmental models, such as prenatal administration of methylazomethanol (MAM) (Lodge and Grace, 2009) display impairments in several cognitive functions, including cognitive control (Kállai et al., 2020). In the same line, models of impaired E/I balance, as the adolescent-ketamine administration model (Becker et al., 2003) display impaired behavioral flexibility (Floresco et al., 2009; Zonneveld et al., 2019). Finally, genetic models that replicate genetic susceptibility, as the transgenic models Dlx5/6^+/–^ and Df(16)A^+/–^, display impairment in working memory, behavioral flexibility and inhibitory control (Sigurdsson et al., 2010; Del Pino et al., 2013; Cho et al., 2015).

**TABLE 1:** Rodent models of SZ.

Principle	Model	Dysfunction in prefrontal interneurons	Cognitive impairment		Prefrontal neurophysiological impairment		
			**Working memory**	**Behavioral flexibility**	**Gama oscillations**	**Theta oscillations**	**Phase-locking of neuronal firing**
Dysregulation of the excitatory/inhibitory balance	Chronic adolescent-ketamine administration	Decreased GABAergic neurotransmission	Yes	Yes	Yes	Yes	(-)
Impaired cortical neurodevelopment	Methylazomethanol administration	Decreased GABAergic neurotransmission	Yes	Yes	Yes	Yes	(-)
Susceptibility genes	Dlx5/6^+/–^	Decreased GABAergic neurotransmission	Yes	Yes	Yes	(-)	(-)
	Deletion in the 22q11.2 region (Df(16)A^+/–^)	Decreased GABAergic neurotransmission	Yes	Yes	No	Yes	Yes
	DISC-1 mutant mice	Reduced firing	Yes	Yes	Yes	(-)	Yes
	Arc/Arg3.1 deficient mice	Decreased GABAergic neurotransmission	No	Yes	Yes	Yes	(-)

Table comparing the evidence of structural and functional prefrontal alterations in diverse rodent models of SZ. “Yes”: reported and affected; “No”: reported and not affected; (-): not reported.

Rodent models of SZ replicate several neurophysiological features found in patients. For example, it have been found a reduced number of PV-INs in the mPFC in the MAM model (Lodge et al., 2009). Similarly, ketamine-treated rats during adolescence showed a reduced expression of GAD67 in PV-INs and a decreased GABAergic neurotransmission (Pérez et al., 2019). Similar impairment in inhibitory synaptic transmission has been observed in genetic transgenic mouse models (Fénelon et al., 2013). These results suggest impaired inhibitory neurotransmission in the mPFC of SZ rodent models (Lewis et al., 2005; Lewis, 2012; Del Pino et al., 2013; Cho et al., 2015; Dienel and Lewis, 2019; Sauer and Bartos, 2022). Cortical GABAergic INs are relevant for the emergence of several cortical oscillations (Buzsáki and Draguhn, 2004). Therefore, it is not surprising that animal models of SZ displays aberrant oscillatory activity in the PFC (Uhlhaas and Singer, 2010; Senkowski and Gallinat, 2015; Hunt et al., 2017). Several of these prefrontal oscillatory alterations observed in SZ patients have been also found in animal models of SZ (Speers and Bilkey, 2021). For example, it have been observed decreased prefrontal gamma oscillations in models of impaired E/I balance (Pinault, 2008; Ahnaou et al., 2017; Ma et al., 2018), in the MAM neurodevelopmental model (Lodge et al., 2009) and the genetic models as Dlx5/6^+/–^, DISC1-mutant mice and Arc/Arg3.1 deficient mice (Cho et al., 2015; Gao et al., 2019; Sauer and Bartos, 2022). Interestingly, baseline gamma oscillation was increased in the mPFC of MAM and Dlx5/6^+/–^ models but was decreased in relationship to behavioral flexibility and inhibitory control (Lodge et al., 2009; Cho et al., 2015). Also theta oscillations in the mPFC are impaired in rodent models of SZ. For example, decreased task-evoked theta oscillations in the mPFC was found in the MAM neurodevelopmental model (Lodge et al., 2009). Similarly, it was found reduced theta power in the mPFC of Arc/Arg3.1 deficient mice (Gao et al., 2019). This impairment in theta has also observed in the long-range coordination of the mPFC with the HPC in several rodent models of SZ (Sigurdsson et al., 2010; Del Pino et al., 2013; Dickerson et al., 2014). Importantly, this impaired synchrony correlated with impairment in cognitive performance (Sigurdsson et al., 2010). Of relevance, synchronization of spiking of prefrontal neurons by prefrontal theta and gamma oscillations is impaired in these rodent models of SZ (Sigurdsson et al., 2010; Sauer and Bartos, 2022). If this impaired oscillatory coordination of prefrontal neurons impact in the emergence of NAs representing relevant cognitive features is still unknown. Interestingly, it has been found disorganization of NAs in the visual cortex of ketamine and Df(16)A^+/–^ models of SZ (Hamm et al., 2017).

The recovery of altered prefrontal oscillation has been considered an opportunity for treatment in SZ patients. However, prefrontal stimulation is SZ patients at gamma frequency produced mixed results (Barr et al., 2011; Hoy et al., 2015; Haller et al., 2020). These differences may rely in the limited spatial, temporal, and cell-type specificity of brain stimulation tools used in patients (Cho and Sohal, 2014). Therefore, optogenetic stimulation in animals models seems a reliable tool to investigate this issue. It has been found that optogenetic stimulation of PV-INs at gamma-frequency in the mPFC recovered the behavioral flexibility observed in rodent models of SZ (Cho et al., 2015; Patrono et al., 2023). This evidence give supports the role of emerging prefrontal activity patterns in SZ.

Altogether the body of work reviewed here describe characteristics of rodent models that implicate alterations of PFC function in impaired cognitive control and compare well with human symptomatology in diseases and during the course of aging that affect cognitive control.

## 4 Concluding remarks and perspectives

In this review, we presented and discuss current bibliographic evidence suggesting that cognitive control is altered in normal cognitive decline and pathological mental conditions. We also presented bibliographic evidence showing that cognitive control of behavior is supported by the PFC, a structure that integrates relevant information for the generation of adaptive behavioral responses in a goal-directed manner. Importantly, from studies in rodent animal models, it is suggested that cognitive control is mechanistically supported by particular neural activity patterns in the mPFC, such as NA and brain oscillations, which originate from different levels of spatio-temporal interactions among neural components. Finally, both in patients and animal models, the evidence suggests a robust relationship between aberrant oscillatory activity and impaired cognitive control of behavior observed in normal cognitive decline and pathological mental conditions, such as those observed during aging, mood disorders and schizophrenia.

The collected evidence from human studies suggests that a common phenomenon across normal cognitive decline and pathological metal conditions is an abnormal oscillatory activity in the PFC associated with impaired cognitive control. However, given that the evaluation of micro- and mesoscale mechanisms underlying cognitive control is difficult to approach in human subjects, the utilization of animal models is crucial for understanding primary cause and, therefore, for the development of efficient therapeutic and preventive strategies. Importantly, research performed in animal models has offered relevant evidence about the neuronal and network mechanisms supporting oscillatory activity in the PFC required for cognitive control. Thus, from research in animal models, we suggest a mechanism that integrates research from human patients with data obtained from animal models (schematic diagram in Figure 1B). Particular etiological factors (congenital, environmental, etc.,) affect the organization of prefrontal neural network and its connectivity with distributed neural populations. This induce local and large-scale impairment in functional connectivity, which is macroscopically manifested as abnormal oscillatory activity in the PFC. Thus, the formation, updating, consolidation and activation of NAs representing relevant cognitive features in the PFC are impaired, hindering the adaptive accommodation of behavioral responses according to internal and environmental conditions. Ultimately, these phenomena manifest as perseverative behaviors, as observed in normal cognitive decline and pathological mental conditions.

Considering that the long-term effectivity of current pharmacological treatment for normal and pathological mental conditions is unclear (Barbui et al., 2009), we propose that upcoming therapeutic strategies should be focused on the re-establishment of prefrontal neural activity patterns, together with their coupling with large-scale structures. Therapeutic strategies based on non-invasive brain stimulation, such as repetitive transcranial magnetic stimulation (rTMS) and transcranial direct current stimulation (tDCS), seems to be a promising choice (Regenold et al., 2022). However, the outcome of these therapeutic strategies is mixed, in part due to a lack of understanding of how rhythmic stimulation interacts with ongoing brain dynamics (Sreekumar et al., 2017). Therefore, understanding of the neural processes involved in the implementation of cognitive control is central to the successful implementation of preventive and therapeutic strategies.

## Author contributions

IN-O: Writing – review and editing, Writing – original draft, Supervision, Funding acquisition, Conceptualization. TD: Writing – review and editing, Writing – original draft, Conceptualization. LC-V: Writing – review and editing, Writing – original draft. NL-Q: Writing – review and editing, Writing – original draft. JU-P: Writing – review and editing, Writing – original draft.
